# Supplementary material for: Research on differential game strategy of debt restructuring supported by government
Source: PLoS One. 2023 Apr 6;18(4):e0284044. doi: 10.1371/journal.pone.0284044 (PMC10079094; doi:10.1371/journal.pone.0284044)
Supplement: S1 Data — (DOCX) [file pone.0284044.s001.docx]

**S1 Data Availability statement**

This paper focuses on the multi-agent equilibrium decision-making problem of debt restructuring by using mathematical modeling method. In the "Example Analysis" part of this manuscript, in order to verify the validity of the results of this paper, the relevant parameters are assigned: 𝜃, *𝜇_M_*, *𝜇_N_*, *α*, *β*, *γ*, *ω*, *ρ*, *ψ_0_*, *K*_0_, 𝜏, *σ*, *φ_M_*, *φ_N_*, *t*. The optimal equilibrium strategy of debt restructuring, the optimal trajectory of debt restructuring coordination, and the sensitivity of the overall profit of debt restructuring system to key parameters under different decision-making scenarios are analyzed more intuitively. The above parameters selected for MATLAB2018 simulation analysis in this paper refers to the research results of Giovanni [1], Amrouche [2] and Liu [3], and combines the relevant data in "China Reform Yearbook" and "Accounting Yearbook of China" to make the parameter setting as practical as possible.

China Reform Yearbook:

<https://202.199.103.219/rwt/CNKI_FULLTEXTDB/https/N3RYM4JPMNYGX4JPN3TYE/knavi/yearbooks/YTRQQ/detail?uniplatform=NZKPT&language=chs>

Accounting Yearbook of China:

<https://202.199.103.219/rwt/CNKI_FULLTEXTDB/https/N3RYM4JPMNYGX4JPN3TYE/knavi/yearbooks/YZGKJ/detail?uniplatform=NZKPT&language=chs>
[1] De Giovanni P. Quality Improvement vs. Advertising support: Which strategy works better for a manufacturer?. European J of Operations Research, 2011, 208(2): 119-130. <https://doi.org/10.1016/j.ejor.2010.08.003>

[2] Amrouche N, Martin-Herran G, Zaccour G. Feedback Stackelberg equilibrium strategies when the private label competes with the national brand. Annals of Operations Research, 2008, 164(1): 79-95. https://doi:10.1007/s10479-008-0320-7

[3] Liu Guowei, Sethi S P, Zhang Jianxiong. Myopic vs. far-sighted behavior in a revenue-sharing supply chain with reference quality effects. International Journal of Production Research, 2016, 54(5-6):1-24. <https://doi.org/10.1080/00207543.2015.1068962>

**S2 Appendix A**

Proof of Theorem 2.

The dynamic random control method is used to solve, after time *t*, the optimal value function of long-term profit of creditors and debt enterprises is:$P_{M}^{B}\left( E_{M} \right)=e^{-\rho t}V_{M}^{B}\left( K \right), P_{N}^{B}\left( E_{N} \right)=e^{-\rho t}V_{N}^{B}\left( K \right)$, $V_{M}^{B}\left( K \right)$ and $V_{N}^{B}\left( K \right)$ fit the HJB equation for all *K*≥0. The HJB equation is shown in formula (20).

$$\rho V_{M}^{B}\left( K \right)=\max_{E_{M}\geq0}\left[ \theta\left( \omega K^{B}+\psi_{0} \right)-\frac{\mu_{M}}{2}\left( E_{M}^{B} \right)^{2}+\varphi_{M}\frac{\mu_{M}}{2}\left( E_{M}^{B} \right)^{2}+\tau\frac{\mu_{M}}{2}\left( E_{M}^{B} \right)^{2}+V_{M}^{B'}\left( K \right)\left( \alpha E_{M}^{B}+\beta E_{N}^{B}-\gamma K \right) \right]$$

$\rho V_{N}^{B}\left( K \right)=\max_{E_{N}\geq0}\left[ \left( 1-\theta\right)\left( \omega K^{B}+\psi_{0} \right)-\frac{\mu_{N}}{2}\left( E_{N}^{B} \right)^{2}+\varphi_{N}\frac{\mu_{N}}{2}\left( E_{N}^{B} \right)^{2}+\sigma\left( E_{N}^{B} \right)^{2}+V_{N}^{B'}\left( K \right)\left( \alpha E_{M}^{B}+\beta E_{N}^{B}-\gamma K \right) \right]$ (A.1)

The optimal strategies of both sides are solved by the first derivative:

$$E_{M}^{B}=\frac{\alpha V_{M}^{B'}\left( K \right)}{\mu_{M}\left( 1-\varphi_{M}-\tau\right)}$$

$E_{N}^{B}=\frac{\beta V_{N}^{B'}\left( K \right)}{\mu_{N}\left( 1-\varphi_{N}-\sigma\right)}$ (A.2)

Substituting (A.2) into (A.1):

$${\rho V}_{M}^{B}\left( K \right)=\left( \theta\omega-\gamma V_{M}^{B'} \right)K+\theta\psi_{0}+\frac{\alpha^{2}{V_{M}^{B'}}^{2}}{{2\mu}_{M}\left( 1-\varphi_{M}-\tau\right)}+\frac{\beta^{2}V_{M}^{B'}V_{N}^{B'}}{\mu_{N}\left( 1-\varphi_{N}-\sigma\right)}$$

${\rho V}_{N}^{B}\left( K \right)=\left[ \left( 1-\theta\right)\omega-\gamma V_{N}^{B'} \right]K+\left( 1-\theta\right)\psi_{0}+\frac{\beta^{2}{V_{N}^{B'}}^{2}}{2\mu_{N}\left( 1-\varphi_{N}-\sigma\right)}+\frac{\alpha^{2}V_{M}^{B'}V_{N}^{B'}}{\mu_{M}\left( 1-\varphi_{M}-\tau\right)}$ (A.3)

According to the analysis of equation (A.3), the solution of HJB equation is shown as follows: assume $V_{M}^{B}\left( K \right)=a_{3}K+b_{3}, V_{N}^{B}\left( K \right)=a_{4}K+b_{4}$, where *a_1_*, *a_2_*, *b_1_*, *b_2_* are constants, and we can get:

$$a_{3}=\frac{\theta\omega}{\rho+\gamma}$$

$$b_{3}=\frac{{\theta\psi}_{0}}{\rho}+\frac{\alpha^{2}\theta^{2}\omega^{2}}{2\rho\mu_{M}\left( 1-\varphi_{M}-\tau\right)\left( \rho+\gamma\right)^{2}}+\frac{\beta^{2}\theta\omega^{2}\left( 1-\theta\right)}{\rho\mu_{N}\left( \rho+\gamma\right)^{2}\left( 1-\varphi_{N}-\sigma\right)}$$

$$a_{4}=\frac{\omega-\theta\omega}{\rho+\gamma}$$

$b_{4}=\frac{\psi_{0}-{\theta\psi}_{0}}{\rho}+\frac{\beta^{2}\left( \omega-\theta\omega\right)^{2}}{2\rho\mu_{N}\left( \rho+\gamma\right)^{2}\left( 1-\varphi_{N}-\sigma\right)}+\frac{\alpha^{2}\theta\omega^{2}\left( 1-\theta\right)}{\rho\mu_{M}\left( \rho+\gamma\right)^{2}\left( 1-\varphi_{M}-\tau\right)}$ (A.4)

Substitute equation (A.4) into equation (A.2) to obtain the equilibrium strategy of creditors and debt enterprises under the decentralized decision-making, such as equation (17); Then the optimal strategy equation (17) is substituted into equation (2) to obtain the optimal trajectory of debt restructuring synergy, as shown in equation (18); Finally, the equation (A.4) is substituted into $V_{M}^{B}\left( K \right)=a_{3}K+b_{3}$ and $V_{N}^{B}\left( K \right)=a_{4}K+b_{4}$ respectively, and then the obtained $V_{M}^{B}$and $V_{N}^{B}$ are substituted into $P_{M}^{B}\left( E_{M} \right)=e^{-\rho t}V_{M}^{B}\left( K \right)$and$P_{N}^{B}\left( E_{N} \right)=e^{-\rho t}V_{N}^{B}\left( K \right)$ respectively, which can further obtain the profits of both parties and the total profit of the system, such as equation (19).
