# Supplementary material for: Research on differential game strategy of debt restructuring supported by government
Source: PLoS One. 2023 Apr 6;18(4):e0284044. doi: 10.1371/journal.pone.0284044 (PMC10079094; doi:10.1371/journal.pone.0284044)
Supplement: S1 File — (DOCX) [file pone.0284044.s002.docx]

This paper focuses on the multi-agent equilibrium decision-making problem of debt restructuring by using mathematical modeling method. In the "Example Analysis" part of this manuscript, in order to verify the validity of the results of this paper, the relevant parameters are assigned: 𝜃, *𝜇_M_*, *𝜇_N_*, *α*, *β*, *γ*, *ω*, *ρ*, *ψ_0_*, *K*_0_, 𝜏, *σ*, *φ_M_*, *φ_N_*, *t*. The optimal equilibrium strategy of debt restructuring, the optimal trajectory of debt restructuring coordination, and the sensitivity of the overall profit of debt restructuring system to key parameters under different decision-making scenarios are analyzed more intuitively. The above parameters selected for MATLAB2018 simulation analysis in this paper refers to the research results of **Giovanni [1], Amrouche [2] and Liu [3],** and combines the relevant data in "**China Reform Yearbook**" and "**Accounting Yearbook of China**" to make the parameter setting as practical as possible.

**China Reform Yearbook:**

<https://202.199.103.219/rwt/CNKI_FULLTEXTDB/https/N3RYM4JPMNYGX4JPN3TYE/knavi/yearbooks/YTRQQ/detail?uniplatform=NZKPT&language=chs>

**Accounting Yearbook of China:**

<https://202.199.103.219/rwt/CNKI_FULLTEXTDB/https/N3RYM4JPMNYGX4JPN3TYE/knavi/yearbooks/YZGKJ/detail?uniplatform=NZKPT&language=chs>
**[1]** De Giovanni P. Quality Improvement vs. Advertising support: Which strategy works better for a manufacturer?. European J of Operations Research, 2011, 208(2): 119-130. <https://doi.org/10.1016/j.ejor.2010.08.003>

**[2]** Amrouche N, Martin-Herran G, Zaccour G. Feedback Stackelberg equilibrium strategies when the private label competes with the national brand. Annals of Operations Research, 2008, 164(1): 79-95. https://doi:10.1007/s10479-008-0320-7

**[3]** Liu Guowei, Sethi S P, Zhang Jianxiong. Myopic vs. far-sighted behavior in a revenue-sharing supply chain with reference quality effects. International Journal of Production Research, 2016, 54(5-6):1-24. <https://doi.org/10.1080/00207543.2015.1068962>
