# Supplementary material for: Research on differential game strategy of debt restructuring supported by government
Source: PLoS One. 2023 Apr 6;18(4):e0284044. doi: 10.1371/journal.pone.0284044 (PMC10079094; doi:10.1371/journal.pone.0284044)
Supplement: S2 File — (DOCX) [file pone.0284044.s003.docx]

**Appendix A：**

Proof of Theorem 2.

The dynamic random control method is used to solve, after time *t*, the optimal value function of long-term profit of creditors and debt enterprises is:$P_{M}^{B}\left( E_{M} \right)=e^{-\rho t}V_{M}^{B}\left( K \right)$, $P_{N}^{B}\left( E_{N} \right)=e^{-\rho t}V_{N}^{B}\left( K \right)$, $V_{M}^{B}\left( K \right)$ and $V_{N}^{B}\left( K \right)$ fit the HJB equation for all *K*≥0. The HJB equation is shown in formula (20).

$$\rho V_{M}^{B}\left( K \right)=\max_{E_{M}\geq0}\left[ \theta\left( \omega K^{B}+\psi_{0} \right)-\frac{\mu_{M}}{2}\left( E_{M}^{B} \right)^{2}+\varphi_{M}\frac{\mu_{M}}{2}\left( E_{M}^{B} \right)^{2}+\tau\frac{\mu_{M}}{2}\left( E_{M}^{B} \right)^{2}+V_{M}^{B'}\left( K \right)\left( \alpha E_{M}^{B}+\beta E_{N}^{B}-\gamma K \right) \right]$$

$\rho V_{N}^{B}\left( K \right)=\max_{E_{N}\geq0}\left[ \left( 1-\theta\right)\left( \omega K^{B}+\psi_{0} \right)-\frac{\mu_{N}}{2}\left( E_{N}^{B} \right)^{2}+\varphi_{N}\frac{\mu_{N}}{2}\left( E_{N}^{B} \right)^{2}+\sigma\left( E_{N}^{B} \right)^{2}+V_{N}^{B'}\left( K \right)\left( \alpha E_{M}^{B}+\beta E_{N}^{B}-\gamma K \right) \right]$ (A.1)

The optimal strategies of both sides are solved by the first derivative:

$$E_{M}^{B}=\frac{\alpha V_{M}^{B'}\left( K \right)}{\mu_{M}\left( 1-\varphi_{M}-\tau\right)}$$

$E_{N}^{B}=\frac{\beta V_{N}^{B'}\left( K \right)}{\mu_{N}\left( 1-\varphi_{N}-\sigma\right)}$ (A.2)

Substituting (A.2) into (A.1):

$${\rho V}_{M}^{B}\left( K \right)=\left( \theta\omega-\gamma V_{M}^{B'} \right)K+\theta\psi_{0}+\frac{\alpha^{2}{V_{M}^{B'}}^{2}}{{2\mu}_{M}\left( 1-\varphi_{M}-\tau\right)}+\frac{\beta^{2}V_{M}^{B'}V_{N}^{B'}}{\mu_{N}\left( 1-\varphi_{N}-\sigma\right)}$$

${\rho V}_{N}^{B}\left( K \right)=\left[ \left( 1-\theta\right)\omega-\gamma V_{N}^{B'} \right]K+\left( 1-\theta\right)\psi_{0}+\frac{\beta^{2}{V_{N}^{B'}}^{2}}{2\mu_{N}\left( 1-\varphi_{N}-\sigma\right)}+\frac{\alpha^{2}V_{M}^{B'}V_{N}^{B'}}{\mu_{M}\left( 1-\varphi_{M}-\tau\right)}$ (A.3)

According to the analysis of equation (A.3), the solution of HJB equation is shown as follows: assume $V_{M}^{B}\left( K \right)=a_{3}K+b_{3}$, $V_{N}^{B}\left( K \right)=a_{4}K+b_{4}$, where *a_1_*, *a_2_*, *b_1_*, *b_2_* are constants, and we can get:

$$a_{3}=\frac{\theta\omega}{\rho+\gamma}$$

$$b_{3}=\frac{{\theta\psi}_{0}}{\rho}+\frac{\alpha^{2}\theta^{2}\omega^{2}}{2\rho\mu_{M}\left( 1-\varphi_{M}-\tau\right)\left( \rho+\gamma\right)^{2}}+\frac{\beta^{2}\theta\omega^{2}\left( 1-\theta\right)}{\rho\mu_{N}\left( \rho+\gamma\right)^{2}\left( 1-\varphi_{N}-\sigma\right)}$$

$$a_{4}=\frac{\omega-\theta\omega}{\rho+\gamma}$$

$b_{4}=\frac{\psi_{0}-{\theta\psi}_{0}}{\rho}+\frac{\beta^{2}\left( \omega-\theta\omega\right)^{2}}{2\rho\mu_{N}\left( \rho+\gamma\right)^{2}\left( 1-\varphi_{N}-\sigma\right)}+\frac{\alpha^{2}\theta\omega^{2}\left( 1-\theta\right)}{\rho\mu_{M}\left( \rho+\gamma\right)^{2}\left( 1-\varphi_{M}-\tau\right)}$ (A.4)

Substitute equation (A.4) into equation (A.2) to obtain the equilibrium strategy of creditors and debt enterprises under the decentralized decision-making, such as equation (17); Then the optimal strategy equation (17) is substituted into equation (2) to obtain the optimal trajectory of debt restructuring synergy, as shown in equation (18); Finally, the equation (A.4) is substituted into $V_{M}^{B}\left( K \right)=a_{3}K+b_{3}$ and $V_{N}^{B}\left( K \right)=a_{4}K+b_{4}$ respectively, and then the obtained $V_{M}^{B}$and $V_{N}^{B}$ are substituted into $P_{M}^{B}\left( E_{M} \right)=e^{-\rho t}V_{M}^{B}\left( K \right)$and$P_{N}^{B}\left( E_{N} \right)=e^{-\rho t}V_{N}^{B}\left( K \right)$ respectively, which can further obtain the profits of both parties and the total profit of the system, such as equation (19).
