# Supplementary material for: Research on differential game strategy of debt restructuring supported by government
Source: PLoS One. 2023 Apr 6;18(4):e0284044. doi: 10.1371/journal.pone.0284044 (PMC10079094; doi:10.1371/journal.pone.0284044)
Supplement: Attachment — Submitted filename: Response to Reviewers.docx [file pone.0284044.s004.docx]

**Responses to the reviewer’s comments**

**Ms. Ref. No.: PONE-D-22-29886**

**Title: Research on Differential Game Strategy of Debt Restructuring Supported by Government**

**Journal: PLOS ONE**

Dear Editor and Reviewers,

Thank you very much for your letter and for the reviewers’ comments concerning our manuscript. The comments are all valuable and very helpful for improving the quality of our paper. We agree with the comments and have made amendments accordingly. The changes made in the manuscript are highlighted in blue in the paper. Our point-by-point response to the reviewers’ comments is as follows:

**Journal Requirements:**

When submitting your revision, we need you to address these additional requirements.

1. Please ensure that your manuscript meets PLOS ONE's style requirements, including those for file naming. The PLOS ONE style templates can be found at
https://journals.plos.org/plosone/s/file?id=wjVg/PLOSOne_formatting_sample_main_body.pdf;
<https://journals.plos.org/plosone/s/file?id=ba62/PLOSOne_formatting_sample_title_authors_affiliations.pdf>

Response: The manuscript has been typeset according to the requirements of the journal.

2. Thank you for stating the following financial disclosure:

"This research was funded by National Social Science Foundation of China, grant number 21BGL110."

Please state what role the funders took in the study. If the funders had no role, please state: "The funders had no role in study design, data collection and analysis, decision to publish, or preparation of the manuscript."
If this statement is not correct you must amend it as needed.

Please include this amended Role of Funder statement in your cover letter; we will change the online submission form on your behalf.

Response: This research was funded by National Social Science Foundation of China, grant number 21BGL110.

**The CRediT authorship contribution statement:**

Liangliang Han: The funders had no role in study design, data collection and analysis, decision to publish, or preparation of the manuscript.

3. In your Data Availability statement, you have not specified where the minimal data set underlying the results described in your manuscript can be found. PLOS defines a study's minimal data set as the underlying data used to reach the conclusions drawn in the manuscript and any additional data required to replicate the reported study findings in their entirety. All PLOS journals require that the minimal data set be made fully available. For more information about our data policy, please see http://journals.plos.org/plosone/s/data-availability.

Response: This paper focuses on the multi-agent equilibrium decision-making problem of debt restructuring by using mathematical modeling method. In the "Example Analysis" part of this manuscript, in order to verify the validity of the results of this paper, the relevant parameters are assigned: 𝜃, *𝜇_M_*, *𝜇_N_*, *α*, *β*, *γ*, *ω*, *ρ*, *ψ_0_*, *K*_0_, 𝜏, *σ*, *φ_M_*, *φ_N_*, *t*. The optimal equilibrium strategy of debt restructuring, the optimal trajectory of debt restructuring coordination, and the sensitivity of the overall profit of debt restructuring system to key parameters under different decision-making scenarios are analyzed more intuitively. The above parameters selected for MATLAB2018 simulation analysis in this paper refers to the research results of **Giovanni [1], Amrouche [2] and Liu [3],** and combines the relevant data in "**China Reform Yearbook**" and "**Accounting Yearbook of China**" to make the parameter setting as practical as possible.

**China Reform Yearbook:**

<https://202.199.103.219/rwt/CNKI_FULLTEXTDB/https/N3RYM4JPMNYGX4JPN3TYE/knavi/yearbooks/YTRQQ/detail?uniplatform=NZKPT&language=chs>

**Accounting Yearbook of China:**

<https://202.199.103.219/rwt/CNKI_FULLTEXTDB/https/N3RYM4JPMNYGX4JPN3TYE/knavi/yearbooks/YZGKJ/detail?uniplatform=NZKPT&language=chs>
**[1]** De Giovanni P. Quality Improvement vs. Advertising support: Which strategy works better for a manufacturer?. European J of Operations Research, 2011, 208(2): 119-130. <https://doi.org/10.1016/j.ejor.2010.08.003>

**[2]** Amrouche N, Martin-Herran G, Zaccour G. Feedback Stackelberg equilibrium strategies when the private label competes with the national brand. Annals of Operations Research, 2008, 164(1): 79-95. https://doi:10.1007/s10479-008-0320-7

**[3]** Liu Guowei, Sethi S P, Zhang Jianxiong. Myopic vs. far-sighted behavior in a revenue-sharing supply chain with reference quality effects. International Journal of Production Research, 2016, 54(5-6):1-24. <https://doi.org/10.1080/00207543.2015.1068962>

China Reform Yearbook:

<https://202.199.103.219/rwt/CNKI_FULLTEXTDB/https/N3RYM4JPMNYGX4JPN3TYE/knavi/yearbooks/YTRQQ/detail?uniplatform=NZKPT&language=chs>

Accounting Yearbook of China:

https://202.199.103.219/rwt/CNKI_FULLTEXTDB/https/N3RYM4JPMNYGX4JPN3TYE/knavi/yearbooks/YZGKJ/detail?uniplatform=NZKPT&language=chs
[1] De Giovanni P. Quality Improvement vs. Advertising support: Which strategy works better for a manufacturer?. European J of Operations Research, 2011, 208(2): 119-130. <https://doi.org/10.1016/j.ejor.2010.08.003>

[2] Amrouche N, Martin-Herran G, Zaccour G. Feedback Stackelberg equilibrium strategies when the private label competes with the national brand. Annals of Operations Research, 2008, 164(1): 79-95. https://doi:10.1007/s10479-008-0320-7

[3] Liu Guowei, Sethi S P, Zhang Jianxiong. Myopic vs. far-sighted behavioursin a revenue-sharing supply chain with reference quality effects. International Journal of Production Research, 2016, 54(5-6):1-24. <https://doi.org/10.1080/00207543.2015.1068962>

**Reviewer #1: The Editor:**

I have reviewed overall manuscript on “Research on Differential Game Strategy of Debt Restructuring Supported by Government”. Following are my comments on the manuscript:

1. Abstract is poorly written and need extensive improvement.

Response: Really thanks for your comment and question. We have changed and improved the abstract as follows:

This paper studies the debt restructuring equilibrium decision problem composed of creditors and debt enterprises with the participation of the government and asset management companies. With the differential game, the dynamic optimization models of debt restructuring under three situations: centralized decision-making, decentralized decision-making, and Stackelberg game after introducing cost-sharing contract are constructed, respectively. The optimal equilibrium strategy of debt restructuring, the optimal trajectory of debt restructuring synergy, and the optimal profit under three decision-making situations are investigated and compared. It is found that the synergy effect and total profit of debt restructuring are the highest under centralized decision-making, and the Stackelberg game is superior to decentralized decision-making, which shows that the cost-sharing contract can achieve the coordination of overall interests, improve the debt restructuring environment, and promote the debt restructuring process. Finally, the sensitivity analysis of relevant parameters is carried out through an example, which verifies the effectiveness of the conclusion and provides the scientific basis for the government and asset management companies to participate in debt restructuring successfully. These were highlighted in the revised manuscript. Thanks again for your question.

2. The very first Paragraph is without any reference.

Response: Thanks for your question and correction. We have made the following changes: The international financial crisis broke out, and the debt dilemma of enterprises became more and more serious. The financial ecological environment continued to deteriorate, and listed enterprises enter the peak of debt restructuring. The existence of a large number of non-performing assets in enterprises leads to the decrease in liquidity and the aggravation of asset loss. It can not bring the expected income to the enterprise, which leads to the decline of the competitiveness of enterprise assets. The market activities and economic competition of enterprises are becoming increasingly fierce, and a good economic level is very important for improving social welfare, which also shows the importance of properly handling enterprise debts [1-4]. Since 2013, the international economic environment has changed with inflection point significance. However, the non-performing assets of Chinese enterprises rise instead of falling, and the enterprises are in a state of stagnation and lack liquidity, so it is difficult to bring expected returns to the enterprises, and the loss of assets is extremely serious. The above problems have led to the decline of the overall asset quality of China enterprises, and further stifled the competitiveness of Chinese enterprises in the international market, causing a series of vicious cycles of enterprises debt difficulties [5-7]. These were highlighted in the revised manuscript.

The relative references have been given as followed:

1. XM Su, M Chen, J Yur-Austin, Y Liu. Restructuring degree roadmaps to improve timely graduation in higher education. International Journal of Educational Management. 2019;34(2):432-449. <https://doi.org/10.1108/IJEM-07-2019-0257>
2 Minhaj ud-Din, Muhammad Azam Khan, Muhammad Tariq. [External Debt - Blessing or Curse: Empirical Evidence from Pakistan](https://ideas.repec.org/a/eco/journ1/2020-04-28.html). [International Journal of Economics and Financial Issues](https://ideas.repec.org/s/eco/journ1.html), Econjournals. 2020;10(4) (2020):235-246. https://doi.org/10.32479/ijefi.10134
3 DY Zhao, L Song, LL. Han. Evolutionary Game Analysis of Debt Restructuring Involved by Asset Management Companies. Complexity. 2022 (2022). <https://doi.org/10.1155/2022/2651538>
4 M Azam, C Emirullah, AC Prabhakar, AQ Khan. The role of external debt in economic growth of Indonesia-A blessing or burden?. Middle-East Journal of Scientific Research. 2013;18(8):1111-1118. https://doi.org/10.5829/idosi.wasj.2013.25.08.11760
5 DK Kim. Do self-serving managers choose Chapter 11 filing over out-of-court restructuring?, Journal of Economics and Finance. 2006;30(1):105-114．

https://doi:10.1007/BF02834278

6 IJ. Kim, K Ramaswamy, S Sundaresan. Does Default risk in coupons affect the valuation of corporate bonds: a contingent claims model. Financial Management. 1993;22(3):117-131．https://doi:10.2307/3665932
7 MA Khan, M Tariq. External Debt and Public Investment: A Case Study of Pakistan. Journal of Managerial Sciences. 14 2020.

Thanks again for your question.

3. The introduction part is very weak and seems like literature review. I suggest improving it according to the title of the study.

Response: Thanks for your suggestion. We have made improvements according to your suggestion. We have made the following changes:

The international financial crisis broke out, and the debt dilemma of enterprises became more and more serious. The financial ecological environment continued to deteriorate, and listed enterprises enter the peak of debt restructuring. The existence of a large number of non-performing assets in enterprises leads to the decrease in liquidity and the aggravation of asset loss. It can not bring the expected income to the enterprise, which leads to the decline of the competitiveness of enterprise assets. The market activities and economic competition of enterprises are becoming increasingly fierce, and a good economic level is very important for improving social welfare, which also shows the importance of properly handling enterprise debts [1-4]. Since 2013, the international economic environment has changed with inflection point significance. However, the non-performing assets of Chinese enterprises rise instead of falling, and the enterprises are in a state of stagnation and lack liquidity, so it is difficult to bring expected returns to the enterprises, and the loss of assets is extremely serious. The above problems have led to the decline of the overall asset quality of China enterprises, and further stifled the competitiveness of Chinese enterprises in the international market, causing a series of vicious cycles of enterprises debt difficulties [5-7].

Debt restructuring is an effective means to solve the financial difficulties of enterprises. Many enterprises have brought new vitality to their development through debt restructuring. As an important economic activity, debt restructuring is one of the important means for enterprises to reduce financial risks and integrate social resources [8, 9]. In recent years, the ratio of enterprises debt restructuring has shown a clear upward trend, which has aroused widespread concern in the economic community [10]. Nowadays, countries worldwide have completed a large number of debt restructuring based on the debt situation of non-performing assets of their enterprises. Sweden, Finland, and other countries have turned their enterprises from losses to profits through debt restructuring. In Sweden, in the early 1990s, the Swedish government took supporting enterprises as the starting point to help banks carry out debt restructuring, set up asset management companies, and dispose of non-performing assets, helping the Swedish banking industry gradually out of the trough. The Finnish government rescued banks and enterprises through debt restructuring, supported the development of small and medium-sized enterprises through the stripping and selling non-performing assets of banks, and finally successfully got out of the debt dilemma of non-performing assets. In China, there are also many successful cases of enterprises successfully disposing of non-performing assets through debt restructuring [8-10]. In recent years, many important enterprises related to the national economy and people's livelihood have been reborn through debt restructuring, such as Sanpower Group and Luckin coffee. These enterprises improve their business development and financial situation through debt restructuring. In view of this, debt restructuring should be the best choice for enterprises when they are deeply mired in non-performing assets and debts. Compared with bankruptcy reorganization, reconciliation and bankruptcy liquidation, debt restructuring can better protect the interests of both creditors and debtors, make both sides achieve win-win and maximize their benefits. Therefore, this has also become a direct reason for Chinese enterprises to choose to implement debt restructuring activities when they are in financial difficulties.

When the enterprise has a debt crisis, from the perspective of creditors, if the debt enterprises choose bankruptcy liquidation, and the creditors require its bankruptcy liquidation to repay the debt, it will generate a lot of sunk costs and lose the interests of creditors. The creditor may agree to the debtor to repay the debt in a way lower than the original debt conditions by agreement, and creditors can recover their claims to a certain extent without causing greater losses. On the other hand, debt enterprises will have the opportunity to continue to operate, restore their hematopoietic capacity, and creditors will also get more benefits. For the debtors, debt restructuring can relieve the pressure of debt repayment, improve their operating function, increase the total profits of the current period, effectively solve the financial difficulties and debt crises of enterprises, maintain social stability, and become possible to reverse losses [5]. Based on this, the government should try its best to help enterprises reduce the debt burden, avoid enterprises using new loans to repay previous loans, falling into a vicious cycle of lending, and formulate effective debt management policies to improve economic productivity [11].

In terms of policy, the Chinese government strongly supports debt restructuring of enterprises in debt distress, increases tax rebate policy, provides tax rebate and tax reduction policy, solves difficulties for enterprises in debt distress, expands the scale of government financing guarantee business, and reduces financing guarantee rate. At the same time, in order to help enterprises tide over difficulties, the government has also increased its procurement efforts from small and medium-sized enterprises to help enterprises increase business volume and alleviate the debt crisis [8]. In terms of finance, accelerate the issuance and use of local government special bonds, and the financial department will revitalize the stock of local government special debt limits according to law, and give better play to the role of special bond funds; Delaying administrative fees, supporting the use of policy development financial instruments, promoting the orderly landing of important projects, and helping enterprises to reduce their burdens. Through debt restructuring, the distressed enterprises with room for rescue will be restructured to relieve the debt pressure of distressed enterprises, retain their development potential and inject operational vitality as much as possible.

At present, some scholars have studied the impact of debt restructuring decisions. Suet et al. [1] believe that reaching a debt restructuring agreement is a decision-making process. The creditor will decide the way of debt renegotiation according to the situation, the government has a certain influence on the bank's personnel appointment and removal and major decisions, the debtor's decision will also affect the debt restructuring process. Mo et al. [12] find that as the strength of political connection increases from weak to strong, the positive impact of debt restructuring on investment turns to a negative impact. Bergström et al. [13] indicated that restricting the priority of secured debt may stimulate debt restructuring, and there is a negative correlation between the security of financial institution lenders and the possibility of debt restructuring. Relevant scholars conduct research on debt pricing [5-9], such as Kim et al. [6] constructed a liquidity default model. Anderson et al. [8] (1996) constructed a strategic debt payment model. Leland et al. [9] constructed default models caused by economic distress. Macas et al. [14] found that the more companies that use debt in price agreements, the more unstable the price agreements are. Acharya et al. [15] (2006) tried to combine strategic debt payment with equity refinancing and introduce equity refinancing to solve the problem of insufficient liquidity, but were limited to establishing a static model.

Based on the above research results, the existing research mainly focuses on the influencing factors and debt pricing of debt restructuring. However, the research results on the scientific decision-making of each participant in the debt restructuring process and the interaction of various strategic influencing factors are rare. The application of mathematical modeling method in multi-agent decision-making of debt restructuring is still in its infancy. This paper studies debt restructuring in a new way, fills the gap in the multi-agent decision-making theory of debt restructuring, tries to improve the debt restructuring system, interprets the multi-agent decision-making problem of debt restructuring from a new perspective, opens up a new path for the multi-agent decision-making research of debt restructuring, optimizes the existing research on debt restructuring, and enriches relevant literature. The debt restructuring process is a complex and dynamic process resulting from the familiar game of all participants. Due to information asymmetry, it is difficult for each participant to achieve a specific equilibrium in a decision, and the interaction between participants is needed to achieve the final equilibrium. The differential game just makes up for the deficiency of the traditional game method. By extending the game theory to continuous time through differential games, each participant can change their strategy in an infinitely short time period, so as to solve a dynamic model of multi-party cooperation or competition in continuous time. Therefore, this paper uses the method of the differential game to construct the debt restructuring game model between creditors and debt enterprises with the participation of the government and asset management companies to help all parties find the Pareto optimal equilibrium decision of system profits.

The contributions of this paper are as follows: (1) This paper studies the balanced decision-making of debt restructuring with multi-agent participation from a dynamic perspective, providing correct theoretical guidance for promoting the success of debt restructuring. (2) From the perspective of game theory, using the method of differential game, this paper constructs the differential game model of debt restructuring between creditors and debt enterprises with the participation of the government and asset management companies. Through the analysis of centralized decision-making, decentralized decision-making and decision-making after the introduction cost-sharing contract, this paper investigates the optimal effort level of participants, the synergistic effect of debt restructuring, the interests of all participants and the overall income of debt restructuring system in three situations, and finds the optimal decision-making mode and mechanism of debt restructuring. (3) This paper fills the blank of debt restructuring in multi-agent decision-making theory, tries to improve the debt restructuring system, interprets the multi-agent decision-making problem of debt restructuring from a new perspective, opens up a new path for the research of debt restructuring multi-agent decision-making, optimizes the existing research of debt restructuring, enriches the theoretical system of debt restructuring, and has an excellent expansion effect on the relevant theories of debt restructuring multi-agent decision-making. (4) The debt restructuring process is inseparable from the participation of the government and asset management companies. This paper studies the problem of government subsidy to debt restructuring parties and the debt preference given by asset management companies. On this basis, it discusses the income distribution mechanism between the two sides of debt restructuring, analyzes the efforts of debt enterprises and the influence of the strategies chosen by creditors on the performance of debt restructuring, and provides the theoretical basis for the government to formulate subsidy policies.

Thanks again for your suggestion.

4. Motivation and objectives of the study need more focus.

Response: Thanks for your suggestion. Through the study of the existing literature, although there have been a lot of studies on the relevant theories and influencing factors of debt restructuring, however, there are few research results on the scientific decision-making of each participant in the process of debt restructuring and the interaction of influencing factors of various strategies, and the application of mathematical modeling in multi-agent decision-making of debt restructuring is still in its infancy. This paper studies debt restructuring with a new method, fills the gap of debt restructuring in the theory of multi-agent decision-making, tries to improve the system of debt restructuring, interprets the problem of multi-agent decision-making of debt restructuring from a new perspective, opens up a new path for the study of multi-agent decision-making of debt restructuring, optimizes the existing research on debt restructuring, and enriches relevant literature. In this paper, the differential game method is used to solve the balanced decision-making problem of debt restructuring with the participation of multi-agents, to find the optimal decision-making mode and mechanism of debt restructuring, to pay attention to the government's subsidies to creditors and debt enterprises, and to discuss the income distribution mechanism between the two sides of debt restructuring, to analyze the influence of the efforts of debt enterprises and the decisions made by creditors on the performance of debt restructuring, and to put forward corresponding countermeasures and suggestions. It will enrich the theoretical debt restructuring system and expand the relevant theories of multi-agent decision-making in debt restructuring.

According to your suggestion, we emphasized the research motivation and purpose of this paper, and supplemented the relevant contents in the manuscript: This paper studies debt restructuring in a new way, fills the gap in the multi-agent decision-making theory of debt restructuring, tries to improve the debt restructuring system, interprets the multi-agent decision-making problem of debt restructuring from a new perspective, opens up a new path for the multi-agent decision-making research of debt restructuring, optimizes the existing research on debt restructuring, and enriches relevant literature. The debt restructuring process is a complex and dynamic process resulting from the familiar game of all participants. Due to information asymmetry, it is difficult for each participant to achieve a specific equilibrium in a decision, and the interaction between participants is needed to achieve the final equilibrium. The differential game just makes up for the deficiency of the traditional game method. By extending the game theory to continuous time through differential games, each participant can change their strategy in an infinitely short time period, so as to solve a dynamic model of multi-party cooperation or competition in continuous time. Therefore, this paper uses the method of the differential game to construct the debt restructuring game model between creditors and debt enterprises with the participation of the government and asset management companies to help all parties find the Pareto optimal equilibrium decision of system profits.

Thanks again for your suggestion.

5. A summary of previous related studies is to be given in one Table form at the end of the Introduction part. Take help of these close related studies:
- External debt - blessing or curse: empirical evidence from Pakistan. International Journal of Economics and Financial Issues.
- External debt and public investment: a case study of Pakistan. Journal of Managerial Sciences,
-The role of external debt in economic growth of Indonesia – A Blessing or Burden?
- Does military expenditure increase external debt? Evidence from Asia. Defence and Peace Economics.
-Estimating the Optimum Level of Debt-Threshold: Empirical Evidence from Pakistan. Pakistan Social Sciences Review.

Response: Thanks again for your suggestion. Thank you for your suggestion. According to your suggestion, we have quoted and supplemented the relevant literature in the introduction of the manuscript, which is highlighted in the revised manuscript.

6. Source at the end of Table needs to be given where needed.

Response: Thanks again for your suggestion. Thank you for your correction. We have quoted relevant documents at corresponding positions in the revised version and put them in the references of the revised version.

The relative references have been given as followed:

https://doi.org/10.32479/ijefi.10134

http://doi.org/10.5829/idosi.wasj.2013.25.08.11760

<https://www.researchgate.net/publication/344045580_External_Debt_and_Public_Investment_A_Case_Study_of_Pakistan>

<http://doi.org/10.35v484/pssr.2021(5-II)77>

http://doi.org/10.1080/10242694.2015.1072371

7. Shed more lights on the discussion on results.

Response: Thanks for your suggestion. We pay more attention to the discussion of the results, as follows:

As an effective method to solve dynamic problems, the differential game is often applied to environmental protection, pollution reduction, pricing and supply chain [19-23], and few scholars apply it to the research of debt restructuring. Based on the existing literature, this paper constructs a differential game model to study the optimal decisions of creditors and debt enterprises under different decision-making situations. In order to more intuitively compare the optimal equilibrium strategies of debt restructuring under the three situations of centralization, decentralization and the introduction of cost-sharing contract, Table 1 lists the parameters of equilibrium strategies.

**Table 1.** **Comparison of differential game equilibrium strategies under different decision-making situations**

| **equilibrium strategy** | **centralized decision-making** | **decentralized decision-making** | **Steinberg decision-making** |
| --- | --- | --- | --- |
| **The effort cost of creditors** | $\frac{\omega\alpha}{\mu_{M}\left( \rho+\gamma\right)\left( 1-\varphi_{M}-\tau\right)}$ | $\frac{\alpha\theta\omega}{\mu_{M}\left( \rho+\gamma\right)\left( 1-\varphi_{M}-\tau\right)}$ | $\frac{\alpha\omega\theta}{\mu_{M}\left( \rho+\gamma\right)\left( 1-\varphi_{M}-\tau\right)}$ |
| **The effort cost of debt enterprises** | $\frac{\omega\beta}{\mu_{N}\left( \rho+\gamma\right)\left( 1-\varphi_{N}-\sigma\right)}$ | $\frac{\omega\beta\left( 1-\theta\right)}{\mu_{N}\left( \rho+\gamma\right)\left( 1-\varphi_{N}-\sigma\right)}$ | $\frac{\beta\omega\left( 1+\theta\right)}{2\mu_{N}\left( \rho+\gamma\right)\left( 1-\varphi_{N}-\sigma\right)}$ |
| **The effort cost shared by creditors for debt enterprises** | — | — | $\frac{\left( 3\theta-1 \right)\left( 1-\varphi_{N}-\sigma\right)}{\theta+1}$ |
| **The effort cost shared by debt enterprises for creditors** | — | — | — |

By comparing the optimal debt restructuring effort level, debt restructuring synergy effect and total profit under the three situations, the following three propositions are obtained.

Proposition 1. compares the results under three kinds of decisions, and we know that: $E_{N}^{A*}>E_{N}^{B*}=E_{N}^{*}$; when $1/3<\theta<1$，$E_{M}^{A*}>{E_{M}^{*}>E}_{M}^{B*}$, $D^{A}>D>D^{B}$， $P_{M}>P_{M}^{B*}$, $P_{N}^{B*}>P_{N}$, $P_{K}^{A*}>P_{K}>P_{K}^{B*}$; when $0<\theta\leq1/3$, $E_{M}^{A*}>{E_{N}^{*}>E}_{N}^{B*}$, $D^{A}>D>D^{B}$, $P_{M}>P_{M}^{B*}$, $P_{N}^{B*}>P_{N}$, $P_{K}^{A*}>P_{K}>P_{K}^{B*}$. That is, under centralized decision-making, the debt restructuring effort level, the total profit of the system and the synergistic effect of debt restructuring are the highest. This shows that centralized decision-making can enhance the enthusiasm of both parties to participate in debt restructuring and promote the total profit of the system. However, both parties should voluntarily implement centralized decision-making and meet constraints. That is, under centralized decision-making, the profits of both parties are higher than those of the other two decision-making modes.

Prove: $E_{N}^{A*}-E_{N}^{B*}=\frac{\omega\alpha\left( 1-\theta\right)}{\mu_{M}\left( \rho+\gamma\right)\left( 1-\varphi_{M}-\tau\right)}>0$, therefore, the same can be proved, $E_{N}^{B*}=E_{N}^{*}$；$E_{M}^{A*}-E_{M}^{B*}=\frac{\theta\omega\beta}{\mu_{N}\left( \rho+\gamma\right)\left( 1-\varphi_{N}-\sigma\right)}>0$, the same can be proved that ${E_{M}^{*}>E}_{M}^{B*}$；$D^{A}-D^{B}=\frac{\left( 1-\theta\right)}{\gamma\mu_{M}\left( \rho+\gamma\right)\left( 1-\varphi_{M}-\tau\right)}+\frac{\theta\omega\beta^{2}}{\gamma\mu_{N}\left( \rho+\gamma\right)\left( 1-\varphi_{N}-\sigma\right)}>0$, therefore, the same can prove that $D>D^{B}$.

Proposition 2. Under the Stackelberg game, the cost-sharing ratio of creditor *M* to debt enterprise *N* is affected by the income ratio of creditors. The cost-sharing ratio of creditors to debt enterprises is affected by the income ratio of creditors. When the creditor's income ratio is $1/3<\theta<1$, *M* shares part of the cost for *N*. At this time, the efforts level of *M* and *N* are higher than those of decentralized decision-making, which shows that the cost-sharing contract plays an incentive role. When the creditor's income ratio is $0<\theta\leq1/3$, *M* does not share the cost of *N*, that is, it has no incentive effect. At this time, the efforts level of *M* and *N* are lower than those of decentralized decision-making.

Proposition 3. Under the three situations, the optimal effort level of both parties and the debt restructuring effort level of creditors and debt enterprises is in direct proportion to the influence coefficient of synergistic effect of debt restructuring, the impact of debt restructuring synergy on total income, the government subsidy rates for *M* and *N* and the asset management companies reduce the effort cost of enterprises and creditors. It is inversely proportional to the discount factor, the natural attenuation rate of synergistic effect and the effort cost coefficient of creditors and debt enterprises. Under decentralized decision-making, the optimal effort level of both parties is also in direct proportion to the distribution ratio of the total proceeds of debt restructuring between creditors and debt enterprises. In the Stackelberg game, the optimal effort level of the creditor is directly proportional to the income ratio of the creditor *M*. The optimal effort level of debt enterprises is directly proportional to the proportion of income obtained by debt enterprises *N.* In the three situations, the total profit of the system is proportional to the influence coefficient of debt restructuring synergy effect on total debt restructuring income, the influence coefficient of the level of efforts of both parties on the synergistic effect of debt restructuring and the government subsidy ratio; it is inversely proportional to the sum of the cost coefficients of both parties of the discount rate and the natural decay rate of the synergistic effect of debt restructuring. This is consistent with the research conclusion of Biglaiser et al. [24], that is, the government tends to use economic flexibility to balance the interests of all parties in debt restructuring.

Proposition 3 shows that when the government subsidy rates *𝞿_M_* and *𝞿_N_* are large and the proportion of preferential strength ratio given by asset management companies is large, the debt restructuring efforts of both parties and the total system profit will increase. Therefore, under centralized decision-making, the cost of debt restructuring can be reduced through government subsidies or incentives from asset management companies. This is consistent with Zabalza's [25] research conclusion, that is, the government needs to update the financial mechanism reasonably to solve the huge debt problem between the main bodies. Under decentralized decision-making, creditors and debt enterprises only pay attention to their respective incomes. The greater the proportion of their own income, the greater the initiative to participate in debt restructuring. Under the Steinberg game, creditors and debtors encourage each other and share costs. However, in order to make the creditor participate in the cost-sharing contract voluntarily, some constraints need to be satisfied, that is, when the condition $1/3<\theta<1$ is satisfied. At this time, the creditor's profit under the cost-sharing contract is greater than that under the decentralized decision-making. The specific profit distribution scheme depends on their negotiating ability, and the cost-sharing agreement needs to be unanimously adopted and strictly implemented by both parties, so as to ensure the optimal profit value under the condition of realizing the cost-sharing contract.

Inference 1. Combining Proposition 1, Proposition 2, Proposition 3, and Table 1 shows that the optimal feedback equilibrium strategies under the three decisions are all time-independent parameters. That is, the optimal feedback equilibrium strategy does not change with time, which is consistent with the actual situation. And from a dynamic point of view, it is verified that under the decision-making situation after the introduction of the cost-sharing contract, the efforts cost of both parties and the total profit of the debt restructuring system have been improved, and reaching the level of centralized decision-making, and achieving long-term win-win cooperation among all parties in debt restructuring, which is consistent with Yulianto's [26] research conclusion, that is, adopting correct policy guidance to maximize the profits of all parties in debt restructuring. When the market is more mature, the operation mode of the debt restructuring system under this decision tends to be stable and the strategy after introducing the cost-sharing contract is feasible in the actual debt restructuring process. The model has good management practical significance, which provides an effective theoretical basis for the successful decision of debt restructuring.

Inference 2. In centralized decision-making, the synergistic effect and total profit of debt restructuring of creditors and debt enterprises are higher than those in decentralized decision-making. The Pareto improvement effect of cost-sharing contract on the total profits of both parties is affected by effort cost coefficient of creditor *μ_M_* and debt enterprise *μ_N_* and the influence coefficients *α* and *β* of the debt restructuring efforts of creditors and debt enterprises on the synergistic effect of debt restructuring. The influence relationship of some parameters will be analyzed in an example.

We have supplemented and highlighted this in the revised manuscript.

Thanks again for your suggestion.

8. Focus more on the policy recommendations.

Response: Thank you very much for your suggestion, we added a section to the manuscript to analyze the policy recommendations.

**Government Subsidy Policy Analysis**

Figs 9 and 10 show the influence of government subsidy rate on the synergistic effect of debt restructuring and the total profit of the system under centralized decision-making.

**Fig 9. Effect of parameter** $\boldsymbol{\varphi}_{\boldsymbol{M}}$ **and** $\boldsymbol{\varphi}_{\boldsymbol{N}}$ **on the synergistic effect of debt restructuring**

**Fig 10. Effect of parameter** $\boldsymbol{\varphi}_{\boldsymbol{N}}$ **and** $\boldsymbol{\varphi}_{\boldsymbol{N}}$ **on total profit of debt restructuring**

This paper analyzes the influence of government support policies on the synergistic effect of debt restructuring between creditors and debt enterprises and the total profit of debt restructuring system, and makes sensitivity analysis on $\varphi_{M}$ and $\varphi_{N}$ under centralized decision, as shown in Figs 9 and 10.

As shown in Fig 9, the synergistic effect of debt restructuring increases with the increase of government subsidies to creditors and debt enterprises, which shows that government support can encourage all subjects of debt restructuring to actively participate in debt restructuring. Therefore, the government can improve the synergistic effect of debt restructuring by increasing the subsidies to creditors and debtors, thus promoting the process of debt restructuring. However, the subsidies of other parameters will have different degrees of influence on the synergistic effect of debt restructuring.

As shown in Fig 10, with the increase of the government's support for debt restructuring, the total profit of the debt restructuring system also increases with the increase of the government's subsidy rate. As the leader in the debt restructuring system, creditors can benefit from government subsidies to debt enterprises while giving them preferential treatment. Therefore, government subsidies can help debt restructuring better coordinate development. This shows that the government can increase the total profit of the system by increasing the degree of subsidies to creditors and debtors, but the subsidy rates to creditors and debtors will have different degrees of influence on the synergistic effect of debt restructuring.

These were highlighted in the revised manuscript.

Thanks again for your suggestion.

9. Highlight main contributions of the study.

Response: Thanks again for your correction. Thank you for your suggestion. According to your suggestion, we emphasized the main contributions of the research in the revised manuscript, as follows: The contributions of this paper are as follows: (1) This paper studies the balanced decision-making of debt restructuring with multi-agent participation from a dynamic perspective, providing correct theoretical guidance for promoting the success of debt restructuring. (2) From the perspective of game theory, using the method of differential game, this paper constructs the differential game model of debt restructuring between creditors and debt enterprises with the participation of the government and asset management companies. Through the analysis of centralized decision-making, decentralized decision-making and decision-making after the introduction cost-sharing contract, this paper investigates the optimal effort level of participants, the synergistic effect of debt restructuring, the interests of all participants and the overall income of debt restructuring system in three situations, and finds the optimal decision-making mode and mechanism of debt restructuring. (3) This paper fills the blank of debt restructuring in multi-agent decision-making theory, tries to improve the debt restructuring system, interprets the multi-agent decision-making problem of debt restructuring from a new perspective, opens up a new path for the research of debt restructuring multi-agent decision-making, optimizes the existing research of debt restructuring, enriches the theoretical system of debt restructuring, and has an excellent expansion effect on the relevant theories of debt restructuring multi-agent decision-making. (4) The debt restructuring process is inseparable from the participation of the government and asset management companies. This paper studies the problem of government subsidy to debt restructuring parties and the debt preference given by asset management companies. On this basis, it discusses the income distribution mechanism between the two sides of debt restructuring, analyzes the efforts of debt enterprises and the influence of the strategies chosen by creditors on the performance of debt restructuring, and provides the theoretical basis for the government to formulate subsidy policies. These were highlighted in the revised manuscript. Thanks again for your suggestion.

Once again, thank you for your constructive comments concerning our manuscript.

**Reviewer #2:**

I am pleased to review the manuscript titled "Research on Differential Game Strategy of Debt Restructuring Supported by Government" in which authors tried to investigate the debt restructuring equilibrium strategy through differential game method. The findings of the study are interesting particularly related to the synergy effect and total profit of debt restructuring under centralized decision-making, and the synergy effect and total profit of debt restructuring after the introduction of cost-sharing. The following comments will help to enhance the quality of the manuscript:

1. The discussion of the findings is missing. Please contextualize your findings with the literature.

Response: Really thanks for your comment and correction. Thank you for your comments and corrections. According to your request, we have added relevant contents in the manuscript, as follows: As an effective method to solve dynamic problems, the differential game is often applied to environmental protection, pollution reduction, pricing and supply chain [19-23], and few scholars apply it to the research of debt restructuring. Based on the existing literature, this paper constructs a differential game model to study the optimal decisions of creditors and debt enterprises under different decision-making situations. In order to more intuitively compare the optimal equilibrium strategies of debt restructuring under the three situations of centralization, decentralization and the introduction of cost-sharing contract, Table 1 lists the parameters of equilibrium strategies.

**Table 1.** **Comparison of differential game equilibrium strategies under different decision-making situations**

| **equilibrium strategy** | **centralized decision-making** | **decentralized decision-making** | **Steinberg decision-making** |
| --- | --- | --- | --- |
| **The effort cost of creditors** | $\frac{\omega\alpha}{\mu_{M}\left( \rho+\gamma\right)\left( 1-\varphi_{M}-\tau\right)}$ | $\frac{\alpha\theta\omega}{\mu_{M}\left( \rho+\gamma\right)\left( 1-\varphi_{M}-\tau\right)}$ | $\frac{\alpha\omega\theta}{\mu_{M}\left( \rho+\gamma\right)\left( 1-\varphi_{M}-\tau\right)}$ |
| **The effort cost of debt enterprises** | $\frac{\omega\beta}{\mu_{N}\left( \rho+\gamma\right)\left( 1-\varphi_{N}-\sigma\right)}$ | $\frac{\omega\beta\left( 1-\theta\right)}{\mu_{N}\left( \rho+\gamma\right)\left( 1-\varphi_{N}-\sigma\right)}$ | $\frac{\beta\omega\left( 1+\theta\right)}{2\mu_{N}\left( \rho+\gamma\right)\left( 1-\varphi_{N}-\sigma\right)}$ |
| **The effort cost shared by creditors for debt enterprises** | — | — | $\frac{\left( 3\theta-1 \right)\left( 1-\varphi_{N}-\sigma\right)}{\theta+1}$ |
| **The effort cost shared by debt enterprises for creditors** | — | — | — |

By comparing the optimal debt restructuring effort level, debt restructuring synergy effect and total profit under the three situations, the following three propositions are obtained.

Proposition 1. compares the results under three kinds of decisions, and we know that: $E_{N}^{A*}>E_{N}^{B*}=E_{N}^{*}$; when $1/3<\theta<1$，$E_{M}^{A*}>{E_{M}^{*}>E}_{M}^{B*}$, $D^{A}>D>D^{B}$， $P_{M}>P_{M}^{B*}$, $P_{N}^{B*}>P_{N}$, $P_{K}^{A*}>P_{K}>P_{K}^{B*}$; when $0<\theta\leq1/3$, $E_{M}^{A*}>{E_{N}^{*}>E}_{N}^{B*}$, $D^{A}>D>D^{B}$, $P_{M}>P_{M}^{B*}$, $P_{N}^{B*}>P_{N}$, $P_{K}^{A*}>P_{K}>P_{K}^{B*}$. That is, under centralized decision-making, the debt restructuring effort level, the total profit of the system and the synergistic effect of debt restructuring are the highest. This shows that centralized decision-making can enhance the enthusiasm of both parties to participate in debt restructuring and promote the total profit of the system. However, both parties should voluntarily implement centralized decision-making and meet constraints. That is, under centralized decision-making, the profits of both parties are higher than those of the other two decision-making modes.

Prove: $E_{N}^{A*}-E_{N}^{B*}=\frac{\omega\alpha\left( 1-\theta\right)}{\mu_{M}\left( \rho+\gamma\right)\left( 1-\varphi_{M}-\tau\right)}>0$, therefore, the same can be proved, $E_{N}^{B*}=E_{N}^{*}$；$E_{M}^{A*}-E_{M}^{B*}=\frac{\theta\omega\beta}{\mu_{N}\left( \rho+\gamma\right)\left( 1-\varphi_{N}-\sigma\right)}>0$, the same can be proved that ${E_{M}^{*}>E}_{M}^{B*}$；$D^{A}-D^{B}=\frac{\left( 1-\theta\right)}{\gamma\mu_{M}\left( \rho+\gamma\right)\left( 1-\varphi_{M}-\tau\right)}+\frac{\theta\omega\beta^{2}}{\gamma\mu_{N}\left( \rho+\gamma\right)\left( 1-\varphi_{N}-\sigma\right)}>0$, therefore, the same can prove that $D>D^{B}$.

Proposition 2. Under the Stackelberg game, the cost-sharing ratio of creditor *M* to debt enterprise *N* is affected by the income ratio of creditors. The cost-sharing ratio of creditors to debt enterprises is affected by the income ratio of creditors. When the creditor's income ratio is $1/3<\theta<1$, *M* shares part of the cost for *N*. At this time, the efforts level of *M* and *N* are higher than those of decentralized decision-making, which shows that the cost-sharing contract plays an incentive role. When the creditor's income ratio is $0<\theta\leq1/3$, *M* does not share the cost of *N*, that is, it has no incentive effect. At this time, the efforts level of *M* and *N* are lower than those of decentralized decision-making.

Proposition 3. Under the three situations, the optimal effort level of both parties and the debt restructuring effort level of creditors and debt enterprises is in direct proportion to the influence coefficient of synergistic effect of debt restructuring, the impact of debt restructuring synergy on total income, the government subsidy rates for *M* and *N* and the asset management companies reduce the effort cost of enterprises and creditors. It is inversely proportional to the discount factor, the natural attenuation rate of synergistic effect and the effort cost coefficient of creditors and debt enterprises. Under decentralized decision-making, the optimal effort level of both parties is also in direct proportion to the distribution ratio of the total proceeds of debt restructuring between creditors and debt enterprises. In the Stackelberg game, the optimal effort level of the creditor is directly proportional to the income ratio of the creditor *M*. The optimal effort level of debt enterprises is directly proportional to the proportion of income obtained by debt enterprises *N.* In the three situations, the total profit of the system is proportional to the influence coefficient of debt restructuring synergy effect on total debt restructuring income, the influence coefficient of the level of efforts of both parties on the synergistic effect of debt restructuring and the government subsidy ratio; it is inversely proportional to the sum of the cost coefficients of both parties of the discount rate and the natural decay rate of the synergistic effect of debt restructuring. This is consistent with the research conclusion of Biglaiser et al. [24], that is, the government tends to use economic flexibility to balance the interests of all parties in debt restructuring.

Proposition 3 shows that when the government subsidy rates *𝞿_M_* and *𝞿_N_* are large and the proportion of preferential strength ratio given by asset management companies is large, the debt restructuring efforts of both parties and the total system profit will increase. Therefore, under centralized decision-making, the cost of debt restructuring can be reduced through government subsidies or incentives from asset management companies. This is consistent with Zabalza's [25] research conclusion, that is, the government needs to update the financial mechanism reasonably to solve the huge debt problem between the main bodies. Under decentralized decision-making, creditors and debt enterprises only pay attention to their respective incomes. The greater the proportion of their own income, the greater the initiative to participate in debt restructuring. Under the Steinberg game, creditors and debtors encourage each other and share costs. However, in order to make the creditor participate in the cost-sharing contract voluntarily, some constraints need to be satisfied, that is, when the condition $1/3<\theta<1$ is satisfied. At this time, the creditor's profit under the cost-sharing contract is greater than that under the decentralized decision-making. The specific profit distribution scheme depends on their negotiating ability, and the cost-sharing agreement needs to be unanimously adopted and strictly implemented by both parties, so as to ensure the optimal profit value under the condition of realizing the cost-sharing contract.

Inference 1. Combining Proposition 1, Proposition 2, Proposition 3, and Table 1 shows that the optimal feedback equilibrium strategies under the three decisions are all time-independent parameters. That is, the optimal feedback equilibrium strategy does not change with time, which is consistent with the actual situation. And from a dynamic point of view, it is verified that under the decision-making situation after the introduction of the cost-sharing contract, the efforts cost of both parties and the total profit of the debt restructuring system have been improved, and reaching the level of centralized decision-making, and achieving long-term win-win cooperation among all parties in debt restructuring, which is consistent with Yulianto's [26] research conclusion, that is, adopting correct policy guidance to maximize the profits of all parties in debt restructuring. When the market is more mature, the operation mode of the debt restructuring system under this decision tends to be stable and the strategy after introducing the cost-sharing contract is feasible in the actual debt restructuring process. The model has good management practical significance, which provides an effective theoretical basis for the successful decision of debt restructuring.

Inference 2. In centralized decision-making, the synergistic effect and total profit of debt restructuring of creditors and debt enterprises are higher than those in decentralized decision-making. The Pareto improvement effect of cost-sharing contract on the total profits of both parties is affected by effort cost coefficient of creditor *μ_M_* and debt enterprise *μ_N_* and the influence coefficients *α* and *β* of the debt restructuring efforts of creditors and debt enterprises on the synergistic effect of debt restructuring. The influence relationship of some parameters will be analyzed in an example.

We have supplemented and highlighted this in the revised manuscript.

Thanks again for your suggestion.

The relative references have been given as followed:

19 XZ Hao, BZ Li. Research on collaborative innovation among enterprises in green supply chain based on carbon emission trading. Science Progress. 2020;103(2). <https://doi.org/10.1177/0036850420916329>
20 ZY Zhang, LY Yu. Dynamic Optimization and Coordination of Cooperative Emission Reduction in a Dual-Channel Supply Chain Considering Reference Low-Carbon Effect and Low-Carbon Goodwill. International journal of environmental research and public health. 2021;18(2):539. <https://doi.org/10.3390/ijerph18020539>
21 Q Hou, JY Sun. Investment strategy analysis of emission-reduction technology under cost subsidy policy in the carbon trading market. Kybernetes. 2020;49(2):252-284. <https://doi.org/10.1108/K-11-2017-0418>
22 D Chen, Y Zhang, X Hong, QF Chen, J Zhang. Non-Cooperative Game and Cooperative Operation of Multi-Level Supply Chain Under Background ofCarbon Emission Reduction. IEEE ACCESS. 2022;10:33015-33025. https://doi.org/10.1109/ACCESS.2022.3156639
23 N Jaakkola. Carbon taxation, OPEC and the end of oil. Journal of Environmental Economics and Management. 2019; 94:101-117. <https://doi.org/10.1016/j.jeem.2019.01.011>
24 Biglaiser G, McGauvran RJ. The effects of debt restructurings on income inequality in the developing world. European Journal of International Relations. 2021;27(3):808–829. https://doi.org/10.1177/13540661211001425
25 Zabalza A. Restructuring proposal for the Spanish regional debt. Investigaciones Regionales–Journal of Regional Research. 2021;(2021/2) 50: 71-103. <https://doi.org/10.38191/iirr-jorr.21.012>
26 Yulianto A. Comparative Analysis and Overview of Indonesian & Global Debt Restructuring Policies Amid the Covid-19 Pandemic. Croatian International Relations Review. 2022;28(89):102-119. https://doi.org/10.2478/CIRR-2022-0006

2. The motivation and rationale of the study needs further attention by the authors.

Response: Thanks for your suggestion. According to your request, we have paid more attention to the motivation and rationale of this study in the revised manuscript.

Through the study of the existing literature, although there have been a lot of studies on the relevant theories and influencing factors of debt restructuring, however, there are few research results on the scientific decision-making of each participant in the process of debt restructuring and the interaction of influencing factors of various strategies, and the application of mathematical modeling in multi-agent decision-making of debt restructuring is still in its infancy. This paper studies debt restructuring with a new method, fills the gap of debt restructuring in the theory of multi-agent decision-making, tries to improve the system of debt restructuring, interprets the problem of multi-agent decision-making of debt restructuring from a new perspective, opens up a new path for the study of multi-agent decision-making of debt restructuring, optimizes the existing research on debt restructuring, and enriches relevant literature. In this paper, the differential game method is used to solve the balanced decision-making problem of debt restructuring with the participation of multi-agents, to find the optimal decision-making mode and mechanism of debt restructuring, to pay attention to the government's subsidies to creditors and debt enterprises, and to discuss the income distribution mechanism between the two sides of debt restructuring, to analyze the influence of the efforts of debt enterprises and the decisions made by creditors on the performance of debt restructuring, and to put forward corresponding countermeasures and suggestions. It will enrich the theoretical debt restructuring system and expand the relevant theories of multi-agent decision-making in debt restructuring.

According to your suggestion, we emphasized the research motivation and purpose of this paper, and supplemented the relevant contents in the manuscript: This paper studies debt restructuring in a new way, fills the gap in the multi-agent decision-making theory of debt restructuring, tries to improve the debt restructuring system, interprets the multi-agent decision-making problem of debt restructuring from a new perspective, opens up a new path for the multi-agent decision-making research of debt restructuring, optimizes the existing research on debt restructuring, and enriches relevant literature. The debt restructuring process is a complex and dynamic process resulting from the familiar game of all participants. Due to information asymmetry, it is difficult for each participant to achieve a specific equilibrium in a decision, and the interaction between participants is needed to achieve the final equilibrium. The differential game just makes up for the deficiency of the traditional game method. By extending the game theory to continuous time through differential games, each participant can change their strategy in an infinitely short time period, so as to solve a dynamic model of multi-party cooperation or competition in continuous time. Therefore, this paper uses the method of the differential game to construct the debt restructuring game model between creditors and debt enterprises with the participation of the government and asset management companies to help all parties find the Pareto optimal equilibrium decision of system profits.

Thanks again for your suggestion.

3. The manuscript is very less reader friendly. The theoretical discussion can be moved to the annexure.

Response: Thanks for your question and suggestion. We have put some contents in the appendix of the revised manuscript, as follows:

Appendix A：

Proof of Theorem 2.

The dynamic random control method is used to solve, after time *t*, the optimal value function of long-term profit of creditors and debt enterprises is:$P_{M}^{B}\left( E_{M} \right)=e^{-\rho t}V_{M}^{B}\left( K \right)$, $P_{N}^{B}\left( E_{N} \right)=e^{-\rho t}V_{N}^{B}\left( K \right)$, $V_{M}^{B}\left( K \right)$ and $V_{N}^{B}\left( K \right)$ fit the HJB equation for all *K*≥0. The HJB equation is shown in formula (20).

$$\rho V_{M}^{B}\left( K \right)=\max_{E_{M}\geq0}\left[ \theta\left( \omega K^{B}+\psi_{0} \right)-\frac{\mu_{M}}{2}\left( E_{M}^{B} \right)^{2}+\varphi_{M}\frac{\mu_{M}}{2}\left( E_{M}^{B} \right)^{2}+\tau\frac{\mu_{M}}{2}\left( E_{M}^{B} \right)^{2}+V_{M}^{B'}\left( K \right)\left( \alpha E_{M}^{B}+\beta E_{N}^{B}-\gamma K \right) \right]$$

$\rho V_{N}^{B}\left( K \right)=\max_{E_{N}\geq0}\left[ \left( 1-\theta\right)\left( \omega K^{B}+\psi_{0} \right)-\frac{\mu_{N}}{2}\left( E_{N}^{B} \right)^{2}+\varphi_{N}\frac{\mu_{N}}{2}\left( E_{N}^{B} \right)^{2}+\sigma\left( E_{N}^{B} \right)^{2}+V_{N}^{B'}\left( K \right)\left( \alpha E_{M}^{B}+\beta E_{N}^{B}-\gamma K \right) \right]$	(A.1)

The optimal strategies of both sides are solved by the first derivative:

$$E_{M}^{B}=\frac{\alpha V_{M}^{B'}\left( K \right)}{\mu_{M}\left( 1-\varphi_{M}-\tau\right)}$$

$E_{N}^{B}=\frac{\beta V_{N}^{B'}\left( K \right)}{\mu_{N}\left( 1-\varphi_{N}-\sigma\right)}$	(A.2)

Substituting (A.2) into (A.1):

$${\rho V}_{M}^{B}\left( K \right)=\left( \theta\omega-\gamma V_{M}^{B'} \right)K+\theta\psi_{0}+\frac{\alpha^{2}{V_{M}^{B'}}^{2}}{{2\mu}_{M}\left( 1-\varphi_{M}-\tau\right)}+\frac{\beta^{2}V_{M}^{B'}V_{N}^{B'}}{\mu_{N}\left( 1-\varphi_{N}-\sigma\right)}$$

${\rho V}_{N}^{B}\left( K \right)=\left[ \left( 1-\theta\right)\omega-\gamma V_{N}^{B'} \right]K+\left( 1-\theta\right)\psi_{0}+\frac{\beta^{2}{V_{N}^{B'}}^{2}}{2\mu_{N}\left( 1-\varphi_{N}-\sigma\right)}+\frac{\alpha^{2}V_{M}^{B'}V_{N}^{B'}}{\mu_{M}\left( 1-\varphi_{M}-\tau\right)}$	(A.3)

According to the analysis of equation (A.3), the solution of HJB equation is shown as follows: assume $V_{M}^{B}\left( K \right)=a_{3}K+b_{3}$, $V_{N}^{B}\left( K \right)=a_{4}K+b_{4}$, where *a_1_*, *a_2_*, *b_1_*, *b_2_* are constants, and we can get:

$$a_{3}=\frac{\theta\omega}{\rho+\gamma}$$

$$b_{3}=\frac{{\theta\psi}_{0}}{\rho}+\frac{\alpha^{2}\theta^{2}\omega^{2}}{2\rho\mu_{M}\left( 1-\varphi_{M}-\tau\right)\left( \rho+\gamma\right)^{2}}+\frac{\beta^{2}\theta\omega^{2}\left( 1-\theta\right)}{\rho\mu_{N}\left( \rho+\gamma\right)^{2}\left( 1-\varphi_{N}-\sigma\right)}$$

$$a_{4}=\frac{\omega-\theta\omega}{\rho+\gamma}$$

$b_{4}=\frac{\psi_{0}-{\theta\psi}_{0}}{\rho}+\frac{\beta^{2}\left( \omega-\theta\omega\right)^{2}}{2\rho\mu_{N}\left( \rho+\gamma\right)^{2}\left( 1-\varphi_{N}-\sigma\right)}+\frac{\alpha^{2}\theta\omega^{2}\left( 1-\theta\right)}{\rho\mu_{M}\left( \rho+\gamma\right)^{2}\left( 1-\varphi_{M}-\tau\right)}$	(A.4)

Substitute equation (A.4) into equation (A.2) to obtain the equilibrium strategy of creditors and debt enterprises under the decentralized decision-making, such as equation (17); Then the optimal strategy equation (17) is substituted into equation (2) to obtain the optimal trajectory of debt restructuring synergy, as shown in equation (18); Finally, the equation (A.4) is substituted into $V_{M}^{B}\left( K \right)=a_{3}K+b_{3}$ and $V_{N}^{B}\left( K \right)=a_{4}K+b_{4}$ respectively, and then the obtained $V_{M}^{B}$and $V_{N}^{B}$ are substituted into $P_{M}^{B}\left( E_{M} \right)=e^{-\rho t}V_{M}^{B}\left( K \right)$and$P_{N}^{B}\left( E_{N} \right)=e^{-\rho t}V_{N}^{B}\left( K \right)$ respectively, which can further obtain the profits of both parties and the total profit of the system, such as equation (19).

Thanks again for your suggestion.

4. Please explain how this work stands out from the literature in the said field. How it can be assist in policy design.

Response: Thank you for your question. According to your request, we explained how this work stands out from the literature in the above fields, as follows: Based on the above research results, the existing research mainly focuses on the influencing factors and debt pricing of debt restructuring. However, the research results on the scientific decision-making of each participant in the debt restructuring process and the interaction of various strategic influencing factors are rare. And the research of domestic and foreign scholars on debt restructuring mainly focuses on the theoretical aspect, and there are still many practical problems to be solved. The application of mathematical modeling method in multi-agent decision-making of debt restructuring is still in its infancy. This paper studies debt restructuring in a new way, fills the gap in the multi-agent decision-making theory of debt restructuring, tries to improve the debt restructuring system, interprets the multi-agent decision-making problem of debt restructuring from a new perspective, opens up a new path for the multi-agent decision-making research of debt restructuring, optimizes the existing research on debt restructuring, and enriches relevant literature.

We added a section to the manuscript to analyze the policy recommendations, as follows:

**Government Subsidy Policy Analysis**

Figs 9 and 10 show the influence of government subsidy rate on the synergistic effect of debt restructuring and the total profit of the system under centralized decision-making.

**Fig 9. Effect of parameter** $\boldsymbol{\varphi}_{\boldsymbol{M}}$ **and** $\boldsymbol{\varphi}_{\boldsymbol{N}}$ **on the synergistic effect of debt restructuring**

**Fig 10. Effect of parameter** $\boldsymbol{\varphi}_{\boldsymbol{N}}$ **and** $\boldsymbol{\varphi}_{\boldsymbol{N}}$ **on total profit of debt restructuring**

This paper analyzes the influence of government support policies on the synergistic effect of debt restructuring between creditors and debt enterprises and the total profit of debt restructuring system, and makes sensitivity analysis on $\varphi_{M}$ and $\varphi_{N}$ under centralized decision, as shown in Figs 9 and 10.

As shown in Fig 9, the synergistic effect of debt restructuring increases with the increase of government subsidies to creditors and debt enterprises, which shows that government support can encourage all subjects of debt restructuring to actively participate in debt restructuring. Therefore, the government can improve the synergistic effect of debt restructuring by increasing the subsidies to creditors and debtors, thus promoting the process of debt restructuring. However, the subsidies of other parameters will have different degrees of influence on the synergistic effect of debt restructuring.

As shown in Fig 10, with the increase of the government's support for debt restructuring, the total profit of the debt restructuring system also increases with the increase of the government's subsidy rate. As the leader in the debt restructuring system, creditors can benefit from government subsidies to debt enterprises while giving them preferential treatment. Therefore, government subsidies can help debt restructuring better coordinate development. This shows that the government can increase the total profit of the system by increasing the degree of subsidies to creditors and debtors, but the subsidy rates to creditors and debtors will have different degrees of influence on the synergistic effect of debt restructuring.

The government subsidy rate and the preferential strength given by asset management companies will affect the process of debt restructuring. When the government support increases, the level of debt restructuring efforts of both sides and the total profits of the system will increase. Therefore, under centralized decision-making, the cost of debt restructuring can be reduced through government subsidies or incentives from asset management companies. The government should strengthen tax reduction for endangered enterprises and lower the threshold of debt restructuring, thus improving the success rate of debt restructuring. Under the Stackelberg game, creditors and debt enterprises encourage each other and share costs with each other The cost-sharing agreement needs to be unanimously approved and strictly implemented by both parties to ensure the optimal profit value under the cost-sharing contract. The research in this paper has a positive guiding significance for the decision-making of the main body of debt restructuring and the policy making of the government.

These were highlighted in the revised manuscript.

Thanks again for your suggestion.

Once again, thank you for your constructive comments concerning our manuscript.

Thanks again for your efforts to our manuscript during the reviewing. It provides us a good opportunity to improve our manuscript, and we have benefited much during the revising. We express our deepest gratitude to you and the reviewers. The current manuscript has been resubmitted to you and we are looking forward to your positive response.

Yours Sincerely,

L. Song
